# Effect of feeding increasing concentrations of rumen-protected carbohydrate on the growth performance and blood and plasma metabolites of receiving feedlot heifers

**DOI:** 10.1093/tas/txag056

**Published:** 2026-05-04

**Authors:** J P Russi, Nicolas DiLorenzo, Alejandro E Relling

**Affiliations:** RUPCA LLC, CA, United States; University of Florida, North Florida Research and Education Center, Mariana, FL, United States; Department of Animal Sciences, The Ohio State University, Wooster, OH, 44691, United States

**Keywords:** Dry matter intake, Glucose, Growing

## Abstract

The objective of this experiment was to evaluate the inclusion of a rumen-protected carbohydrate (RPC) on growth performance and plasma metabolites in growing beef heifers. Crossbred heifers (*n* = 135; 136 ± 14 kg) were used in a 63-d experiment. Heifers were blocked by initial body weight (BW), placed into 15 pens (9 heifers/pen), and assigned randomly to 1 of 3 treatments 0, 0.5, and 1.0% of RPC. Animals were fed 82.3% of a basal diet (38.8% corn silage, 41.5% dry rolled corn, 2% mineral-vitamin premix on a dry matter basis) and 17.7% supplement. The non-processed supplement or RPC supplement composition (DM basis) were 58.1% soybean meal, 38.9% soluble carbohydrates, 2% urea, and 1% mineral salt. The non-processed supplement or RPC supplement consisted of the same ingredients, differing in the processing of the carbohydrate (i.e., protected or not from ruminal degradation). For the 0, 0.5, and 1% RPC treatments, the ratio of supplement to RPC was 1:0, 1:1, and 0:1, respectively. Body weight was measured on d 0, 21, 42, and 63. Pen dry matter intake (DMI) was measured weekly from d 7 to 63. Back fat on the 12^th^ rib (BF) was measured at d -21 and 63. Blood samples were taken on d -21, 21, 42, and 63 from jugular vein prior morning feeding and analyzed for blood glucose concentration and plasma insulin, urea, and non-esterified fatty acids (NEFA) concentrations. Data were analyzed as a randomized complete block design with repeated measures using a mixed model of SAS (9.4). Treatment × day interaction (*P* ≤ 0.02) was observed for DMI, ADG, and G: F. Heifers on treatment RPC0.5 had the least DMI (*P* < 0.05) and the greatest G: F (*P* < 0.05). No differences (*P* ≥ 0.34) were observed in the concentrations of blood glucose, plasma insulin, plasma NEFA, plasma urea, or BF on d 63. Feeding 8.85% of RPC (treatment RPC0.5) improved G: F through lesser DMI without altering ADG, blood, or plasma metabolites.

## Teaser

Feeding rumen-protected carbohydrates to growing heifers has a dose-dependent effect; where a low dose (0.5% on a dry matter basis) decreases feed intake but increases feed efficiency. A greater dose (1% on a dry matter basis) increases body weight gain.

## Introduction

The receiving period for feedlot cattle is crucial for the later growth performance of the animal. This phase is characterized by multiple stressors associated with environmental transition and dietary change. This phase has been associated with a decrease in dry matter intake (DMI) and growth performance (Fluharty and Loerch 1995). Due to the decreased DMI, a receiving diet has to provide a greater concentration of protein and energy to meet the calf requirement (Fluharty and Loerch 1996; NASEM 2016), compared with growing and finishing diets. However, in the beef nutrient requirement models (NASEM 2016), there is no clear indication of any improvement in protein utilization based on amino acids profile or interaction of the type of protein and energy source. Several experiments report that the amount of energy in the diet influences protein deposition in growing animals (Gerrits et al. 1996; Titgemeyer 2003). The increase in efficiency in protein retention is obtained when net energy intake increases and amino acids are not limiting for growth (Awawdeh et al. 2006; Schroeder et al. 2007). Previous experiments have reported an increase in protein retention when carbohydrates are post-ruminally infused; therefore, the carbohydrates do not suffer rumen fermentation (Asplund et al. 1985; Orskov et al. 1999).

Non-fiber carbohydrates are rapidly and almost entirely fermented in the rumen; therefore, to achieve an increase in post-ruminal absorption of glucose, the sugar has to be protected from rumen fermentation. Recently, beef (Russi et al. 2019) and dairy (McCarthy et al. 2020) cattle have been fed with a rumen-protected soluble carbohydrate (**RPC**). In beef cattle, the response to supplementation with the RPC is dose and weather (heat) dependent, with an increase in animal growth during periods of high temperature humidity index values in animals fed 500 g of RPC/d (Russi et al. 2019). Supplementation with RPC in dairy cattle during the transition period increased plasma glucose concentration and decreased plasma non-esterified fatty acids (**NEFA**) and biomarkers of inflammation (haptoglobin and lipopolysaccharide binding protein) concentrations, with no effect on milk production (McCarthy et al. 2020). However, we are not aware of the use of RPC in receiving diets of feedlot cattle.

Consequently, feeding energy as glucose absorbed in the intestine could benefit protein deposition, which may promote growth in growing cattle. We hypothesize that feeding **RPC** will improve growth performance in early weaned feedlot calves. The objective of the present experiment was to evaluate the effect of feeding increasing concentrations of RPC on the growth performance and blood and plasma metabolites of receiving feedlot heifers.

## Materials and methods

### Facilities

The experiment was carried out in a commercial feedlot in Argentina, in the province of Buenos Aires (34°43ʹ14 S long: 63°05ʹ08 W). Fifteen dirt floor pens (12 × 15 m) with 9 animals each were used for the experiment. Water troughs were shared between 2 pens. All animal procedures were approved by the Animal Care and Use Committee from Veterinary College (Energy metabolism in beef cattle, April 4^th^, 2011; #04012011), La Plata National University, and followed the guidelines recommended in the Guide for the Care and Use of Agricultural Animals in Agricultural Research and Teaching (FASS 2010).

### Animals and treatments

One hundred and thirty-five crossbred heifers (Angus × Hereford) with an average initial body weight (**BW**) of 136 ± 14 kg (mean and standard deviation) were used in an experiment that lasted for 63 d. The heifers were blocked by BW and assigned to 1 of 3 treatments, which were evenly distributed in 5 pens per treatment with 9 heifers per pen. Heifers were fed a basal diet once a day and top dressed with a non-processed supplement or RPC-supplement (Table 1). Feed delivery was managed using slick bunk management (Pritchard and Bruns 2003), feed was offered once a day at 800, 1 h after the feed bunk was read. Treatments were RPC0, RPC0.5, and RPC1.0; where the heifers received a ratio of non-processed supplement and RPC supplement at 1:0, 1:1, and 0:1, respectively, which corresponds to concentrations of 0, 32.31, and 64.63 g of protected glucose/kg of DM. Heifers were fed 82.3% of a basal diet (% DM; Table 1) plus 17.5% supplement. The non-processed supplement and RPC supplement consisted of the same ingredients (58.1% of soybean meal, 38.9% of dextrose, and 2.8% of urea on a dry matter basis), differing in the processing of the carbohydrate (i.e., protected or not from ruminal degradation; Patent N U.S. 8, 507,025 B2). Based on the glucose protection described previously (Patent N U.S. 8,507,025 B2), the goal was to feed 0, 190, or 380 g of protected dextrose for RPC0, RPC0.5, and RPC1.0, respectively, based on an estimated DMI of 5.88 kg/d based on the feed intake equation of the latest Nutrient Requirements of Beef Cattle (NASEM 2016). The diet was formulated to meet requirements for crude protein (CP; at a 1 kg/d of ADG) and meet or exceed mineral and vitamin requirements (NASEM 2016). The composition of the diet that was fed to the animals was (DM basis) 38.8% corn silage, 41.5% dry rolled corn, 2% minerals and vitamins, and 17.7% supplement, or RPC or a combination of both (Table 1), depending on the treatment group. As described previously (Russi et al. 2019; Patent N U.S. 8,507,025 B2), the carbohydrate portion of the supplement was protected because of a Maillard reaction process, which makes the resulting sugar-amino complex minimally affected by ruminal fermentation (Van Soest 1994; Kostyukovsky and Marounek 1995).

**Table 1.** Chemical composition of the final diet, containing increasing doses of rumen-protected carbohydrates in diets fed to heifers.

Ingredient	% dry matter basis
** Corn Silage**	38.8
** Dry rolled corn**	41.5
** Supplement or RPC^1^**	17.7
** Vitamins and minerals^2^**	2.0
**Nutrient composition (% DM basis)**	
** DM**	65.10
** CP**	12.01
** ADF**	19.01
** NDF**	32.73
** EE**	3.41
** Ashes**	6.96

1 The supplement and RPC differed on the processing of the carbohydrate (i.e., protected or unprotected of ruminal degradation). It contains 58.1% soybean meal, 38.9% dextrose, 2.8% urea, and 1.2 mineral salts (63.1% NaHCO_3_, 10.8% K_2_HPO_4_, 8.1% KH_2_PO_4_, 18.0% NaOH).

2 Minerals and vitamins concentration of the premix: CaCO_3_ 32.1%, MgO 3.2%, NaCl 3.2%, CoCO_3_ 6.17 ppm, CuSO_4_ 555.5 ppm, Ca(IO_3_)_2_ 16.2 ppm, MnO 972.2 ppm, Na_2_SeO_3_ 2.3 ppm, ZnSO_4_ 1620.3 ppm, Monensin 0.12%, Vitamin A 69400 UI, Vit E 925 UI.

Individual feed ingredient samples were collected weekly and composited for dry matter content and nutrient composition analysis at the end of the experiment. Feed samples were analyzed for DM (60°C for 48 h), acid and neutral detergent fiber (Ankom Technology methods 5 and 6, respectively; Ankom Technology, Fairport, NY), CP (method 930.15; AOAC 1997), ether extract (method 2; Ankom Technology, Fairport, NY), and total ash (600°C for 12 h).

### Animal growth performance

Heifers were weighed 15 d before starting the experiment, when they started receiving the basal diet, and were blocked according to weight into 5 blocks. Heifers within each block were assigned randomly to 3 treatments, 5 pens per treatment, and 9 animals per pen. From the day of the BW until the start of the experiment, heifers were fed the same basal diet that was used for the rest of the experiment. Animals were weighed on d 0, 21, 42, and 63, always during the morning prior to the morning feeding. Dry matter intake was measured from d 1 onwards once the adaptation period was finished; the refusal was weighed on d 7, 14, 21, 28, 35, 42, 49, 56, and 63, 7 Back-fat on the 12^th^ rib (BF) was measured by ultrasound (Pie Medical mod. Aquila. Transductor 3.5 mhtz) on d -21 and 63.

### Blood samples

Blood samples were taken from the jugular vein before morning feeding on d -1, 21, 42, and 63 from 4 randomly selected animals in each pen. Blood was collected in tubes containing EDTA (1.6 mg/mL of blood). Samples were maintained in a cooler until collection was finished. Blood samples were then centrifuged for 20 min at 1000 × *g* to obtain plasma, which was immediately frozen and stored at -20°C until analysis. A drop of blood was used to measure glucose with a glucometer (Optimum Xceedt, ABBOTT Lab Argentina) on d 0, 21, 42, and 63. Plasma insulin concentration was analyzed on d 21 and 63 with a radioimmunoassay as described previously by Díaz-Torga et al. (2001). The minimum detectable concentration was 0.05 ng/mL. Intraassay sample coefficient of variation was of 8%. Plasma nonesterified fatty acids concentration was measured on d 0, 21, 42, and 63, using the protocol described by Randox labs (FA 115 Randox Laboratories Ltd). The minimum detectable concentration was 72 m*M*, and the coefficients of variation intra and inter samples were 7.48% and 23%, respectively. Plasma urea concentration was measured on d 0, 21, 42, and 63 using the protocol described by Wiener Lab (Rosario, Santa Fe. etc. 2R UREA Color). The minimum detectable concentration of urea was 0.02 g/L, The intra- and inter-assay coefficient of variation were 9.7% and 11%, respectively.

### Statistical analysis

Data were analyzed as a randomized complete block design with repeated measures, using the MIXED procedure of SAS (9.4). The model included treatment, day, and treatment × day interaction as fixed variables and block and pen within block (to define pen as the experimental unit) as random variables. The most appropriate covariance structure was chosen as having the lowest Akaike Information Criterion. The first-order autoregressive covariance structure was used for plasma variables and compound symmetry for the growth performance variables. The SLICE option of SAS was used to separate means when the interaction *P*-value was ≤ 0.05. Post hoc comparison for main effect differences was set by a Fisher protected T-test, using the PDIFF option of SAS. Back fat at 12^th^ rib on d 63 was analyzed using the initial value on d -21 as a covariate. Differences were considered at *P*-value ≤ 0.05, and tendencies at *P*-value > 0.05 and *P*-value ≤ 0.10. Data is presented as least square means and standard error of the mean (SEM).

## Results

**Figure 1.**
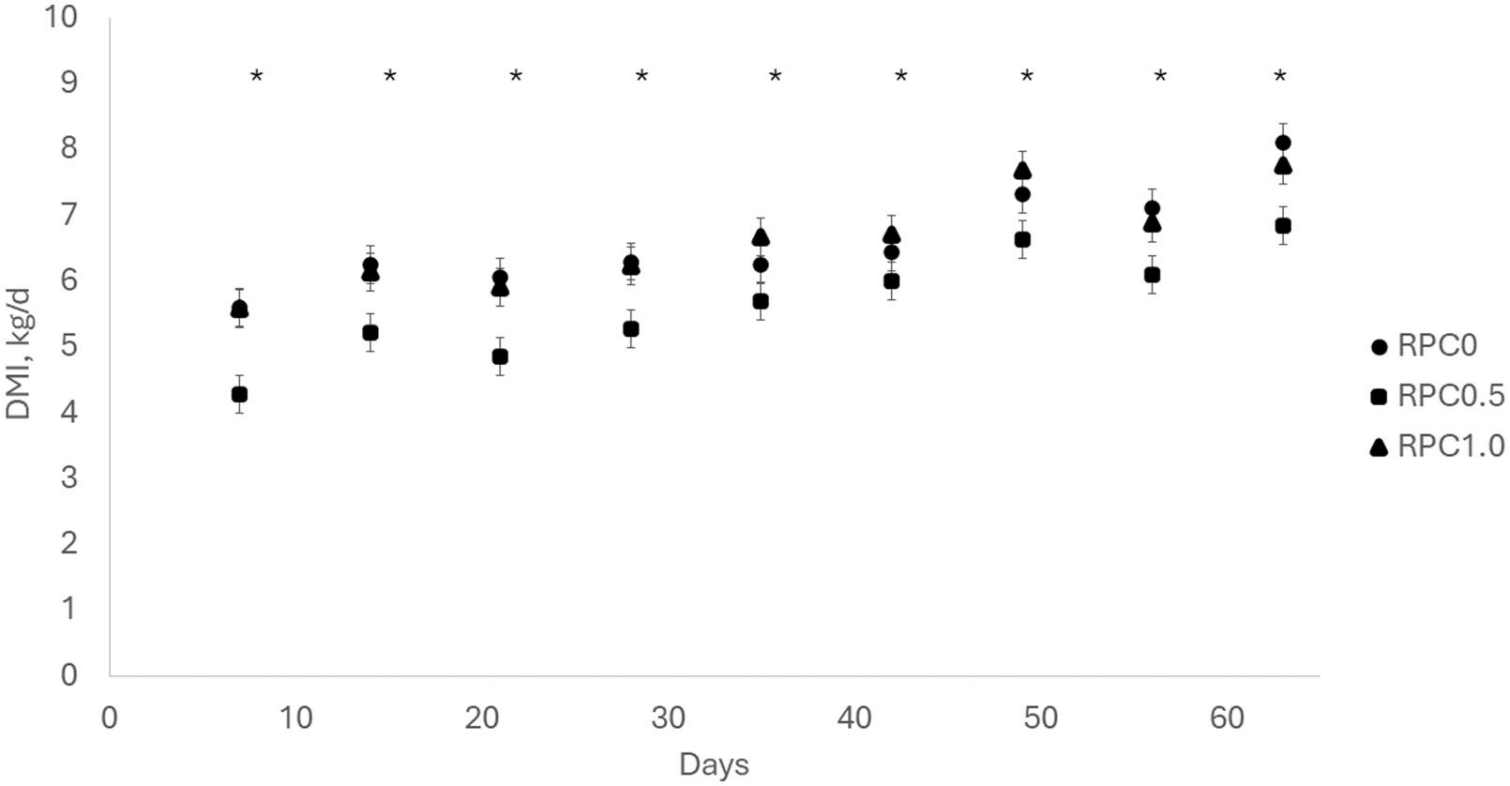
Effect of feeding increasing doses of rumen-protected carbohydrates (RPC at doses of 0, 32.31, and 64.63 g of glucose/kg DM on the RPC0, RPC0.5, and RPC1.0, respectively) on dry matter intake (DMI) in early weaned heifers. Data is presented as least square means and SEM. *P*-value for the treatment by time interaction < 0.01; * *P* < 0.001 using the SLICE option of SAS. Heifers on RPC0.5 had a lesser (*P* ≤ 0.05) DMI than the ones on RPC0 and RPC1 on d 7, 14, 21, 49, 56, and 63. heifers on RPC0.5 had a similar (*P* ≥ 0.36) DMI than the ones on RPC0 and lesser (*P* ≤ 0.05) DMI than the ones on RPC1 on d 28, 35 and 42. There were no differences (*P* ≥ 0.22) on DMI between heifers on RPC0 and RPC1.0.

**Figure 2.**
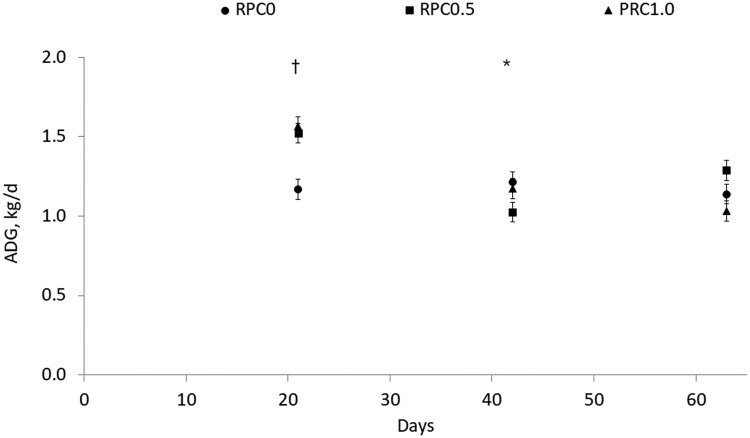
Effect of feeding increasing doses of rumen-protected carbohydrates (RPC at doses of 0, 32.31, and 64.63 g of glucose/kg DM on the RPC0, RPC0.5, and RPC1.0, respectively) on average daily gain (ADG) in early weaned heifers. Data is presented as least square means and SEM. *P*-value for the treatment by time interaction < 0.01; * *P* value < 0.05, † *P* value < 0.001 using the SLICE option of SAS. Heifers in RPC0 had a different (*P* ≤ 0.01) ADG on d 21 compared with RPC0.5 and RPC1.0. Heifers in RPC0.5 had a different (*P* ≤ 0.04) ADG on d 42 compared with RPC0 and RPC1.0.

**Figure 3.**
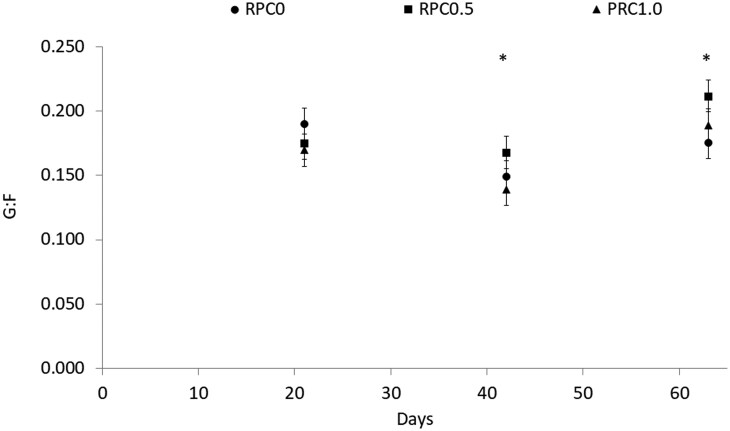
Effect of feeding increasing doses of rumen-protected carbohydrates (RPCat doses of 0, 32.31, and 64.63 g of glucose/kg DM on the RPC0, RPC0.5, and RPC1.0, respectively) on feed conversion efficiency (G: F) in early weaned heifers fed with diets low in protein concentration. Data is presented as least square means and SEM. *P*-value for the treatment by time interaction = 0.05; * *P* value = 0.04 using the SLICE option of SAS. No difference was observed on day 21 (*P* ≥ 0.15) for G: F. On day 42, heifers in RPC0.5 had a greater (*P* = 0.05) G: F than RPC1.0; with heifers in RPC0 in between both (*P* ≥ 0.18). On day 61, RPC0.5 had a greater (*P* = 0.02) G: F than RPC0; with heifers in RPC1 in between both (*P* ≥ 0.11).

There was a treatment × day (*P* < 0.01, Table 2, Fig. 1) interaction for DMI. Heifers on RPC0.5 had a lesser (*P* ≤ 0.05) DMI than the ones on RPC0 and RPC1 on d 7, 14, 21, 49, 56, and 63. heifers on RPC0.5 had a similar (*P* ≥ 0.36) DMI than the ones on RPC0 and lesser (*P* ≤ 0.05) DMI than the ones on RPC1 on d 28, 35 and 42. There were no differences (*P* ≥ 0.22) on DMI between heifers on RPC0 and RPC1.0 (Fig. 1). There was a treatment × time interaction for BW (*P* < 0.01; Table 2). There were no differences (*P* ≥ 0.92) in BW at the beginning of the experiment; however, as the days of feed progressed, heifers in RPC1.0 were heavier than the ones on RPC0.5 (*P* < 0.05), with heifers in RPC0 having an intermediate BW. This difference in BW was observed on d 21 of the experiment and maintained until the end of the experiment. There was a treatment × day interaction difference for average daily gain (*P* < 0.01; Table 2; Fig. 2), heifers in RPC0 had a lesser (*P* ≤ 0.01) ADG on d 21 compared with RPC0.5 and RPC1.0. Heifers in RPC0.5 had a lesser (*P* ≤ 0.04) ADG on d 42 compared with RPC0 and RPC1.0. There was a treatment × day interaction for gain-to-feed ratio (G: F; *P* = 0.05; Table 2; Fig. 3). No difference was observed on day 21 (*P* ≥ 0.15) for G: F. On day 42, heifers in RPC0.5 had a greater (*P* = 0.05) G: F than RPC1.0; with heifers in RPC0 in between both (*P* ≥ 0.18). On day 61, RPC0.5 had a greater (*P* = 0.02) G: F than RPC0; with heifers in RPC1 in between both (*P* ≥ 0.11).

**Table 2.** Effect of feeding increasing doses of rumen-protected carbohydrates (RPC) on dry matter intake (DMI), body weight (BW), average daily gain (ADG), feed conversion efficiency (G: F) rate and thickness of back fat measured on the 12^th^ rib (BFT) measured on d 84 in growing heifers fed low protein diets.

	Treatments^1^				*P*-values		
Item	RPC0	RPC0.5	RPC1.0	SEM	Trt	day	Trt × day
**Pens/heifers**	5/45	5/45	5/45				
**DMI, kg/d**	6.78^a^	5.82^b^	6.75^a^	0.062	< 0.01	< 0.01	< 0.01
**Initial BW, kg**	136.6	136.3	136.3	7.41	0.04	< 0.01	< 0.01
**Final BW, kg**	236.1^ab^	232.8^a^	237.0^b^				
**ADG, kg**	1.18	1.13	1.19	0.027	0.21	< 0.01	< 0.01
**G: F, kg/kg**	0.170	0.185	0.166	0.0124	0.04	< 0.01	0.05
**BF^2^, mm**	55.50	56.26	55.26	1.093	0.80	−	−

1 Rumen-protected carbohydrates (RPC) fed at 0, 0.5, and 1% of the diet dry matter.

2 Back fat thickness on the 12^th^ rib was measured by ultrasound.

No differences were observed on BF on d 63 (*P* = 0.68; Table 2; Fig. 3). No differences (*P* ≥ 0.36) due to treatment or treatment x time interaction were observed for any of the blood or plasma metabolites measured (Table 3).

**Table 3.** Effect of feeding increasing doses of rumen-protected carbohydrates (RPC) on blood glucose concentration and plasma insulin, non-esterified fatty acids (NEFA), and urea concentrations in growing heifers fed low protein diets.

	Treatments^1^				*P-*Values		
Item	RPC0	RPC0.5	RPC1.0	SEM	Trt	day	Trt × day
**Pens/heifers**	5/20	5/20	5/20				
**Glucose, mg/dL**	90.16	91.58	91.19	3.520	0.91	<0.01	0.92
**Insulin, ng/mL**	0.264	0.263	0.236	0.0527	0.82	0.02	0.72
**NEFA, mM**	200.8	187.9	181.3	18.74	0.54	<0.01	0.42
**Urea, g/L**	0.149	0.134	0.148	0.0103	0.36	<0.01	0.39

1 Rumen-protected carbohydrates (RPC) fed at 0, 0.5, and 1% of the diet dry matter.

## Discussion

In the current experiment, we observed differences in growth performance where the supplementation with RPC1.0 increased final BW compared with RPC0.5, but RPC0.5 decreased DMI (during the entire feeding period) but improved G: F compared with RPC0 and RPC1 for days 61 and 42, respectively. The treatments were designed to be iso-nitrogenous and iso-energetic, but different in ruminal degradability. The feed ingredients of all treatments were the same, but rumen degradability of protein and energy was different because of the process of making the RPC. In RPC0, all the ingredients of the supplement consumed by the animal were available for rumen degradation, which might enhance the production of short-chain fatty acids (SCFA) and microbial protein. As described previously (Patent N U.S. 8,507,025 B2), the protection from rumen degradation of the carbohydrate in the RPC is 75%. Therefore, the RPC1.0, with the full dose of RPC, the rumen availability was the least. The RPC0.5 treatment has half the benefit of both treatments, partially enhancing ruminal production with substrates for microbial growth and some of the glucose and protein to be absorbed in the small intestine (Russi et al. 2011).

Protein concentration averaged 12% in all treatments; this concentration is less than what is required for growing animals that averaged a weight of 136 kg when the trial started (NASEM 2016), which, based on the DMI and growth rate of the current experiment, would recommend diets between 14 and 16% CP concentration. In our experiment, the range of DMI was from 5.89 to 6.86 kg, which represented an average CP intake of 0.719 kg/d, 71 g less than that recommended by the nutrient requirements of beef cattle (0.790 kg/d, NASEM 2016) for animals with similar ADG as obtained in our experiment (1.15 kg/d). Furthermore, if we compare specifically each treatment, heifers fed RPC0.5 ate the least CP, 0.148 kg/d less than the recommended (18.6% less). On the other hand, heifers on RPC0 and RPC1.0 ate 0.042 kg/d (5.3% less than recommended) and 0.046 kg/d (5.8% less than recommended), respectively. The difference in CP consumption between treatments was due to the difference in DMI.

Most of the experiments that supplied glucose to the cattle were through infusion and DMI was reported (Kreikemeier and Harmon 1995; Schroeder et al. 2006a). In our experiment, cattle fed RPC0.5 have the least DMI. It is not clear why these differences exist between treatments, but considering these diets, DMI may be regulated by a chemostatic mechanism, such as the absorption of glucose (Van Soest 1994). Based on our results in measured circulating metabolites and hormones, we do not consider that the heifers are the chemostatic metabolites that play a role in the regulation of DMI; however, it is possible that a different absorption of glucose could be affecting DMI by regulation of the secretion of gut peptides (Relling and Reynolds 2008). Despite no differences in blood glucose concentration, it is possible that the absorbed glucose is different. Several experiments on transition cows a reduced DMI was observed when glucose was infused into the abomasum (Larsen and Kristensen 2009; Nichols et al. 2016). This inhibition in DMI could be due to the hepatic oxidation theory, which states a regulatory effect of glucose or glucose precursors metabolized in the liver (Allen et al. 2009). Nonetheless, we would have expected that the greater dose of protected glucose would have a greater effect on DMI inhibition. Another point to consider is that the treatment RPC0.5 provided half a dose of protected glucose and protein and half the dose of ruminally degradable sugar and protein, which might benefit microbial function in the rumen.

Body weight also registered differences between treatments over time; heifers fed RPC0.5 finished the experiment with the lightest body weights of the 3 treatments, which does not surprise and is consistent with the lesser DMI reported by this treatment. Regarding the treatment × day interaction for ADG, differences among treatments during the first 21 d may reflect dietary adaptation, as heifers fed RPC0 exhibited greater ADG during this period. In contrast, during the second period, heifers receiving RPC0.5 and RPC1.0 showed greater ADG. This pattern may indicate a more rapid ruminal adaptation to the RPC0 diet relative to the other 2 treatments during the initial 21 d (Russi et al. 2019), followed by compensatory growth in heifers fed RPC0.5 and RPC1.0 during the subsequent period (Fluharty and Loerch 1995; 1996). Importantly, these differences in ADG appear to be primarily influenced by variations in DMI, independent of differences in G: F.

The 12th rib BF and the rate at which BF was deposited did not differ between treatments. We would have expected differences between treatments regarding this issue in favor of heifers fed RPC0 because this is the treatment that produces more ruminal fermentation, enhancing the production of short-chain fatty acids, particularly acetic, which is a precursor of subcutaneous fat (Smith and Crouse 1984).

We did not observe differences in blood and plasma metabolite concentrations or insulin concentrations between treatments. Maximal doses used in our experiment were similar to those used in other glucose infusion (360 to 380 g/d; Schroeder et al. 2006b). Schroeder et al. (2006b) observed an increase in plasma glucose concentration when glucose was post-ruminally infused. The difference between the results from Schroeder et al. (2006b) and the current experiments might be that our diets contained the same amount of soluble carbohydrates, but with a different site of digestion.

In conclusion, the use of rumen-protected carbohydrates can impact animal growth performance in a time- and dose-dependent manner. The inclusion rate of 0.5% of RPC in the diet decreased DMI and BW; while increasing efficiency, mainly in the first 21 days. The inclusion of 1% RPC in the diet resulted in a similar final body weight; however, the ADG of the 2 treatments differed over time, with the greatest differences occurring during the first 42 days of the experiment, and a difference in feed efficiency on the first 21 days of the experiment. The mechanism of the difference regulation might be due to the availability of different nutrients in the rumen and/or how much is post-ruminally absorbed, but more research needs to be conducted to have a clearer understanding of the effect of rumen-protected carbohydrates in ruminants.

## CRediT statement

J.P. Russi (Conceptualization, Formal analysis, Funding acquisition, Methodology, Resources, Writing—original draft, Writing—review & editing), Nicolas DiLorenzo (Conceptualization, Methodology, Supervision, Writing—review & editing), and Alejandro E. Relling (Conceptualization, Formal analysis, Funding acquisition, Methodology, Project administration, Resources, Supervision, Writing—review & editing).

## Abbreviations

BF: Back fat thickness
ADG: Average daily gain
G: F: Gain to feed ratio
DMI: Dry matter intake
RPC: Rumen-protected carbohydrates
